# Spondylodiscitis Occurring after Diagnostic Lumbar Puncture: A Case Report

**DOI:** 10.1155/2013/843592

**Published:** 2013-02-13

**Authors:** Mehmet Sabri Gürbüz, Mehmet Zafer Berkman

**Affiliations:** Department of Neurosurgery, Haydarpaşa Numune Research and Educational Hospital, Tıbbiye Caddesi, No. 40, Üsküdar, 34668 İstanbul, Turkey

## Abstract

Spondylodiscitis is a rare disease which is generally seen after long-term epidural catheterization. However, spondylidiscitis developing after diagnostic lumbar puncture is very rare. Early diagnosis has a crucial role in the management of the disease and inclines the morbidity rates. However, the diagnosis is often delayed due to the rarity and insidious onset of the disease usually presenting with low back pain which has a high frequency in the society. If it is diagnosed early before development of an abscess requiring surgery or neurological deficit, it responds to antimicrobial therapy quite well. We report 66-year-old male case of spondylodiscitis developing after diagnostic lumbar puncture. The patient was treated with antimicrobial therapy. After antimicrobial therapy, findings of spondylodiscitis were completely resolved and no recurrence was seen in the period of 9-month followup.

## 1. Introduction

Spondylodiscitis is characterized by vertebral osteomyelitis, spondylitis, and discitis. Diagnosis is made with the combination of clinical, radiological, and laboratory findings. Patients present with persistent low back pain, fever, or neurological findings [1, 2]. MRI has high sensitivity and specificity in diagnosis of spondylodiscitis [2, 3] and can reveal signs of spondylodiscitis in even very early stages [3, 4]. Spondylodiscitis responds to antimicrobial therapy well if diagnosed early before the development of neurological deficit and requirement of surgical intervention [2, 5, 6]. We reported a rare case of iatrogenic spondylodiscitis developing after diagnostic lumbar puncture. The patient was diagnosed early and successfully treated with antimicrobial therapy.

## 2. A Case Report

**Figure 1:**
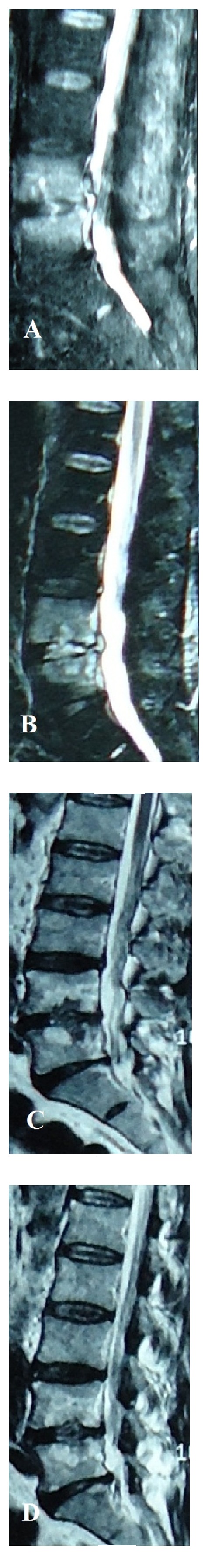
(A) Discitis and osteomyelitis are seen on this T2-weighted MR image of the lumbar spine which demonstrates infective destruction of the L4-5 disk space with the adjacent L4 and L5 vertebral bodies. (B) T2-weighted MR image of the lumbar spine demonstrates diskitis and osteomyelitis persisting despite 4-week treatment of ceftazidime and vancomycine. (C) Eight weeks after the treatment of teikoplanin. T2-weighted MR image of the lumbar spine demonstrates significant resolution in signs of discitis and osteomyelitis. (D) Nine months after the diagnosis. T2-weighted MR image of the lumbar spine demonstrates no recurrence of discitis or osteomyelitis.

 66-years-old male patient was admitted with 3-month history of gait disturbance, urinary incontinence, and mental decline. In cranial CT and MRI scans normal pressure hydrocephalus was suspected, and diagnostic lumbar puncture was done three times every other day. Patient complained of low back pain after the third puncture. Systemic examination was unremarkable. There was no fever. Laboratory examination revealed elevated erythrocyte sedimentation rate (80 mm/h) and C-reactive protein level (3.6 mg/L) with 9.5 × 10^9^/L white blood cells. Lumbar MRI scan was done, and lumbar spondylodiscitis was detected (Figure 1(A).

 Ceftazidime (6000 mg/day) and vancomycin (2000 mg/day) were given for 4 weeks. The blood and CSF cultures were negative. In lumbar MRI scan done after 4 weeks of ceftazidime and vancomycine treatment, signs of spondylodiscitis were persisting (Figure 1(B)), and infection markers were still high (erythrocyte sedimentation rate: 60 mm/h, C-reactive protein: 4.8 mg/L, and white blood cells: 9.1 × 10^9^/L).

Antimicrobial therapy was replaced by teikoplanin (200 mg/day). After 8-week treatment of teikoplanin, infection markers declined to normal ranges, and the patient improved clinically. Lumbar MRI scan revealed significant improvement in signs of spondylodiscitis (Figure 1(C)), and antimicrobial treatment was ended. No recurrence of spondylodiscitis was seen in lumbar MRI done nine months after the time of diagnosis (Figure 1(D)). 

## 3. Discussion

Iatrogenic spondylodiscitis may occur as complication of lumbar disc surgery, epidural catheterization, laser discectomy, percutaneous lumbar nucleotomy, lumbar puncture, and discography [6–12]. Spondylodiscitis seen after diagnostic lumbar puncture, as seen in our case is very rare. 

The diagnosis of spondylodiscitis is usually delayed due to high frequency of low back pain beside the rarity and insidious onset of the disease. When the diagnosis is delayed the mortality and morbidity increase [2, 13]. Jensen et al. reported the rate of correct diagnosis at first presentation as only 5% [14]. In our case, diagnosis of spondylodiscitis was made with the clinical, laboratory, and radiological findings soon after the patient complained of low back pain.

Predisposing factors for spondylodiscitis are foci of local infections, remote infections, AIDS, alcohol use, chronic renal failure, diabetes mellitus, intravenous drug use, malignancy, history of spinal surgery, lumbar catheterization, lumbar puncture, and history of spinal trauma [9, 11]. In 20% of cases there is no predisposing factor [15]. The predisposing factors of our case were history of three serial lumbar punctures and presence of uncontrolled type-2 diabetes mellitus. 

 Spondylodiscitis usually presents with fever, low back pain, local tenderness, neurological deficit, or high infection markers [6–8, 14, 16]. Spondylodiscitis should be suspected when patients present with fever together with low back pain, and clinical, laboratory and radiological evaluations should be done [2, 3, 9]. In our case there were no fever, local tenderness, or neurological deficit. Due to presence of low back pain, high infection markers, and history of repeated lumbar punctures, lumbar MRI scan was done, and spondylodiscitis was diagnosed.

MRI is the most commonly preferred imaging method with the 96% sensitivity and 93% specificity rates due to its superiority to show soft tissues, epidural area, and disc space [2–4]. MRI showed signs of infection successfully in very early stage in our case.

Spondylodiscitis may result from hematogenous spread, direct inoculation, and inoculation from nearby infective tissues [6, 8, 10, 17]. The rate of direct inoculation is 25–30% [2, 4, 11]. Kindler et al. [12] emphasized that clinically silent epidural hematoma developing after lumbar puncture creates entrance point for microorganisms facilitating spondylodiscitis. In our case the level of lumbar puncture and spondylodiscitis was the same, possibly indicating direct inoculation. 

The interval between microbial inoculation and occurrence of clinical manifestations of spondylodiscitis is from 2 to 6 weeks [10, 13]. Early diagnosis is very important because the delay in diagnosis increases the mortality and morbidity rates [13]. Colmenero et al. reported mortality rate as 0–11% which generally results from sepsis [18]. The diagnosis of our case was made 10 days after the first lumbar puncture.

Spondylodiscitis may be bacterial, fungal, parasitic, or mycobacterial [1, 2, 9, 11, 19, 20]. The most frequent pathogen is *staphylococcus aureus* which is isolated in 30–50% of nontuberculosis spondylodiscitis cases [13, 14, 21]. For correct diagnosis blood cultures should be performed because in 60% of spondylodiscitis and epidural abscess cases blood culture is positive [16]. In our case no microorganism was detected in blood culture.

 Antimicrobial therapy is a very important issue in management of spondylodiscitis, whether or not surgical intervention is required. Successful results are achieved with appropriate antibiotics given in sufficient dose and duration. Pathogen-specific antibiotherapy should be given parenterally for 6–12 weeks [2, 5]. After sufficient antibiotic treatment recurrence rates are 0–4% [11, 17]. Clinical recovery is achieved in early phases of treatment, while radiological recovery may be delayed [3]. After antimicrobial therapy clinical and radiological recovery was achieved, and no recurrence was detected in nine-month followup of our case.

In our case, spondylodiscitis may have been resulted from direct inoculation due to nonoptimized sterile conditions. For this reason, optimal sterile conditions should be accomplished especially when serial lumbar punctures will be performed. In any clinical suspect, spondylodiscitis should be diagnosed early, and treatment should begin as soon as possible considering satisfying response of spondylodiscitis to antimicrobial therapy in cases of early diagnosis.
